# Reduced expression of miR-146a in human bronchial epithelial cells alters neutrophil migration

**DOI:** 10.1186/s13601-019-0301-8

**Published:** 2019-11-27

**Authors:** Anet Kivihall, Alar Aab, Jerzy Soja, Krzysztof Sładek, Marek Sanak, Alan Altraja, Bogdan Jakiela, Grazyna Bochenek, Ana Rebane

**Affiliations:** 1 Institute of Biomedicine and Translational Medicine, University of Tartu, Ravila 14B, 50414 Tartu, Estonia; 2 Department of Internal Medicine, Jagiellonian University Medical College, Krakow, Poland; 3 Department of Pulmonary Medicine, University of Tartu, Tartu, Estonia; 4 Lung Clinic of Tartu University Hospital, Tartu, Estonia

**Keywords:** Airway epithelium, HBEC, Endotype, microRNA, miR-146a

## Abstract

**Background:**

The role of miRNAs in the pathogenesis and determining the phenotypes of asthma is not fully elucidated. miR-146a has been previously shown to suppress inflammatory responses in different cells. In this study, we investigated the functions of miR-146a in human bronchial epithelial cells (HBECs) in association with neutrophilic, eosinophilic, and paucigranulocytic phenotypes of asthma.

**Methods:**

Bronchial brushing specimens and brochial mucosal biopsy samples were collected from adult patients with asthma and from age- and gender-matched non-asthmatic individuals. The expression of miR-146a in bronchial brushing specimens, bronchial biopsy tissue sections or cultured primary bronchial epithelial cells was analyzed by RT-qPCR or by in situ hybridization. The expression of direct and indirect miR-146a target genes was determined by RT-qPCR or ELISA. The migration of neutrophils was studied by neutrophil chemotaxis assay and flow cytometry. For statistical analysis, unpaired two-way Student’s *t* test, one-way ANOVA or linear regression analysis were used.

**Results:**

Reduced expression of miR-146a was found in bronchial brushing specimens from asthma patients as compared to non-asthmatics and irrespective of the phenotype of asthma. In the same samples, the neutrophil attracting chemokines IL-8 and CXCL1 showed increased expression in patients with neutrophilic asthma and increased IL-33 expression was found in patients with eosinophilic asthma. Linear regression analysis revealed a significant negative association between the expression of miR-146a in bronchial brushings and neutrophil cell counts in bronchoalveolar lavage fluid of patients with asthma. In bronchial biopsy specimens, the level of miR-146a was highest in the epithelium as determined with in situ hybridization. In primary conventional HBEC culture, the expression of miR-146a was induced in response to the stimulation with IL-17A, TNF-α, and IL-4. The mRNA expression and secretion of IL-8 and CXCL1 was inhibited in both stimulated and unstimulated HBECs transfected with miR-146a mimics. Supernatants from HBECs transfected with miR-146a had reduced capability of supporting neutrophil migration in neutrophil chemotaxis assay.

**Conclusion:**

Our results suggest that decreased level of miR-146a in HBECs from patients with asthma may contribute to the development of neutrophilic phenotype of asthma.

## Background

Asthma affects up to 300 million people worldwide [1]. Patients with asthma suffer from bronchoconstriction and increased mucus production in the airways that results in symptoms like coughing, wheeze and chest tightness [2, 3]. As asthma is a highly heterogeneous disease, attempts have been made to define asthma phenotypes and/or endotypes on the basis of involved immune cells or molecular processes, respectively [4–6]. As one option, this allows categorization of asthmatics into eosinophilic, neutrophilic, mixed granulocytic and paucigranulocytic asthma phenotypes [4, 5]. The eosinophilic phenotype is characterized by the presence of eosinophils in the lung, is frequently associated with increased Th2 responses, involvement of type 2 innate lymphoid cells (ILC2) and increased cytokines IL-4, IL-5 and IL-13 [7, 8]. Patients with paucigranulocytic asthma tend to have fewer overall immune cells in the airways, as measured in the bronchoalveolar lavage fluid (BALF) [4, 5]. In neutrophilic asthma, neutrophil chemoattractants are secreted by bronchial epithelial cells and fibroblasts, for example in response to IL-17 secretion from Th17 cells or ILC3 [9, 10]. Independent from phenotype, airways of all asthma patients are influenced by chronic inflammation, which also leads to the changes in genes affecting cell proliferation, such as HBEGF and FGF2 and eventually, airway remodeling may take place [1–3].

Inhaled glucocorticosteroids represent hallmark of asthma management, often together with other controller medicines that include β_2_-agonists, theophylline, antileukotrienes and anticholinergics [11–13]. In addition, several biologics have become available recently for severe asthma and more will be introduced in the future [6, 14]. However, because these treatment options are not always applicable and occasionally fail to prevent asthma exacerbations, there is still a need for better understanding of the molecular background of asthma to achieve more effective treatment [15, 16].

miRNAs are short endogenous RNAs capable of regulating gene expression through binding their target mRNA via partial complementarity, which leads to inhibition of the translation or mRNA degradation [17–19]. The majority of protein coding genes in human cells and most biological processes, including innate and adaptive immune responses influencing the development of asthma, are regulated by miRNAs [20, 21]. Previous studies have shown that miR-146a has important role in the suppression of inflammatory responses in different cell types [22–24] and therefore is involved in modulation of immune responses. In line with that, we have previously shown that miR-146a inhibits inflammatory responses in human primary keratinocytes and in a mouse model of atopic dermatitis [25, 26]. One of the main target genes of miR-146a is IL-1 receptor-associated kinase 1 (IRAK1) [27], which suppression leads to reduced activity of the nuclear factor (NF)-κB pathway and inhibition of numerous pro-inflammatory chemokines, including C–X–C Motif Chemokine Ligand (CXCL)1 and IL-8 [26, 28]. A recent genome-wide association study of a broad allergic disease phenotype (GWAS; n = 360,838) identified *MIR3142*–*MIR146A* as a shared risk loci of asthma, hay fever and atopic dermatitis [29]. The capacity of miR-146a to inhibit the expression of pro-inflammatory chemokines IL-8 and CCL5 [28], to augment the anti-inflammatory effect of glucocorticosteroids [30] in human lung alveolar epithelial cell line A549, and to inhibit neutrophil elastase-induced gene MUC5AC in immortalized human bronchial epithelial cell line 16HBE [31] has been formerly shown. Very recently, the increased expression of miR-146a in cultured commercial human airway or bronchial epithelial cells (HBECs) was reported [30]. However, no data about miR-146a expression in airway epithelial cells of asthma patients is available, as well as the potential influence of miR-146a on the development or contribution to the heterogeneity of asthma has not been studied before.

Here we analyzed the expression of miR-146a and selected genes either known to be influenced by miR-146a or associated with Th2 or Th1 responses in brush biopsy specimens from patients with asthma. In addition, the expression regulation and function of miR-146a in the modulation of molecular processes associated with asthma was studied in primary conventional HBEC cultures. Our results reveal that reduced expression of miR-146a in HBECs may lead to cellular processes that skew immune responses towards neutrophilic one and thus contribute to the development of neutrophilic phenotype of asthma.

## Materials and methods

### Patient samples

Bronchial brushing samples used for RNA isolation were obtained from 30 adult patients with asthma in stable phase and from 11 age- and gender-matched non-asthmatic individuals from the Department of Pulmonology, the Jagiellonian University Medical College (Krakow, Poland). The diagnosis of asthma was made according to Global Initiative for Asthma (GINA) guidelines. Asthma patients were categorized into eosinophilic, neutrophilic and paucigranulocytic inflammatory phenotypes based on the number of granulocytes present in BALF. Bronchial mucosal biopsy samples for in situ hybridization were collected from 3 adult patients with asthma and 2 non-asthmatic individuals from the Tartu University Hospital (Tartu, Estonia). The study was approved by the Research Ethics Committees of the Jagiellonian University Medical College and the University of Tartu. Each participant provided written informed consent. Additional information regarding recruited individuals is provided in Table 1 and Additional file 1.

**Table 1:** Clinical characteristics of studied patients

Characteristic	All asthmatics	Eosinophilic^a^ phenotype	Neutrophilic^b^ phenotype	Paucigranulocytic^c^ phenotype	Controls
n, (%)	30 (100)	11 (36.7)	7 (23.3)	12 (40.0)	11 (100)
Gender: male/female	16/14	4/7	5/2	7/5	7/4
Age (y), mean ± SD	45.6 ± 13.1	39.8 ± 9.5	48.1 ± 18.7	49.3 ± 11.3	52.2 ± 16.6
Duration of asthma (y), mean ± SD	15.0 ± 16.3	8.3 ± 8.0	13.9 ± 7.9	21.8 ± 22.7	NA
FEV_1_ % predicted	81.8 ± 24.0	86.6 ± 22.7	74.2 ± 32.9	81.9 ± 20.1	111.2 ± 9.6
FEV_1_ % reversibility	7.2 ± 7.2	6.5 ± 5.4	6.2 ± 5.7	8.4 ± 9.5	3.0 ± 3.5
Positive skin prick test with common allergens n, (%)	15 (50.0)	4 (36.4)	4 (57.1)	7 (58.3)	NA
Severity of asthma, median (IQR)^d^	4 (1–5)	4 (2–4)	4 (2–5)	4 (1–5)	NA
Inhaled glucocorticosteroids (%, average dose μg, [range])	96.6, 969 (0–4000)	100, 940 (250–2000)	100, 929 (500–2000)	91.6, 1020 (0–4000)	0
Systemic glucocorticosteroids n, (%)	7 (23.3)	1 (9.1)	4 (57.1)	2 (16.7)	0

*NA* not applicable/available

^a^Eosinophilic (E), > 2% of eosinophils and < 3% of neutrophils in BALF

^b^Neutrophilic (N), < 2% eosinophils and > 3% neutrophils in BALF

^c^Paucigranulocytic (P), < 2% of eosinophils and < 3% neutrophils in BALF

^d^IQR, interquartile range; asthma severity stages according to GINA (Global Initiative for Asthma) classification

### Cell culture, stimulation and transfection of HBECs

HBECs were isolated from bronchial biopsies by initial short-term pronase (Roche, Basel, Switzerland) and DNase (Sigma-Aldrich, USA) digestion. In total, cell lines were isolated from 1 asthma and 4 control subjects. Primary HBECs were cultured as monolayer in supplemented BEGM™ (Bronchial Epithelial Cell Growth Medium, Lonza, Basel, Switzerland) medium. For stimulation, IFN-γ (final concentration 20 ng/ml, eBiosciences, USA), TNF-α (20 ng/ml, Biolegend, USA), IL-17A (10 ng/ml, Peprotech, UK), IL-22 (20 ng/ml, Peprotech, UK) and IL-4 (40 ng/ml, Peprotech, UK) were used. For transfections, miRIDIAN microRNA Mimic Negative Control #1 (Dharmacon™, USA) or miRIDIAN microRNA hsa-miR-146a-5p mimic (Dharmacon™, USA) were transfected using MIRFECT (RNAexact, Estonia) according to manufacturer’s protocol. Additional information can be found in Additional file 1.

### RNA isolation, cDNA synthesis and RT-qPCR

For RNA isolation Qiazol (Qiagen, Germany) and Total RNAzole out Mini kit (A&A Biotechnology, Poland) were used according to the manufacturer’s instructions. To analyze miRNA expression, either TaqMan^®^ MicroRNA Assays (Life Technologies, California, USA) and 5× HOT FIREPol^®^ Probe qPCR Mix Plus (ROX) (Solis BioDyne, Tartu, Estonia) or miScript II RT Kit, miScript SYBR Green PCR Kit and Hs_miR-146a_1 miScript Primer Assay (cat. MS00003535) by Qiagen were used according to the manufacturer’s protocols. For normalization let-7a and ΔΔCt calculation were used. For mRNA RT-qPCR, cDNA was synthesized using oligo-dT (TAG Copenhagen, Denmark), RevertAid Reverse Transcriptase (Thermo Scientific) followed by qPCR with 5× HOT FIREPol EvaGreen qPCR Supermix (Solis BioDyne, Estonia). Target gene expression was normalized to EEF1A1 expression using ΔΔCt calculation. Additional information is provided in Additional file 1.

### ELISA and neutrophil chemotaxis assay

Supernatants from HBECs transfected with miRNA mimics and stimulated with cytokines were used for ELISA and neutrophil chemotaxis assay. ELISA MAX™ Deluxe Set (BioLegend, 431504) for IL-8 and human CXCL1/GRO alpha DuoSet ELISA (R&D Systems, DY275-05) were used according to the manufacturers’ instructions. For neutrophil chemotaxis assay, 4 × 10^5^ of primary human neutrophils were seeded on ThinCert cell culture inserts (3-μm pore size) (Greiner Bio-One, Kremsmünster, Austria) placed in 24 well plate containing the supernatants from HBECs. 60 min after incubation at 37 °C, the number of migrated neutrophils was analyzed by using BD LSRFortessa (BD Biosciences, USA) cell analyzer. More detailed information is provided in Additional file 1.

### In situ hybridization (ISH)

ISH was performed using 10 μm sections of frozen bronchial mucosal biopsy specimens. Using miRCURY LNA miRNA ISH Buffer Set (FFPE), hsa-miR-146a-5p miRCURY LNA miRNA Detection probe (cat. YD00619856) and control probe miRCURY scrambled ISH 49 °C (cat. YCD0074470-BCG) were used according to the manufacturer’s (Qiagen) protocol. Densitometry analysis of staining in in situ hybridization images were performed with ImageJ software. Additional information is provided in Additional file 1.

### Statistical analysis

For visualization and statistical analysis, GraphPad Prism 5 (GraphPad Software Inc, USA) and unpaired two-way Student’s t-test, One-way ANOVA or linear regression analysis were used. The results were considered significant at *P < 0.05; **P < 0.01; ***P < 0.001. For heatmapping, online software Morpheus (https://software.broadinstitute.org/morpheus/) was used.

## Results

### The expression of miR-146a is reduced in bronchial brush specimens and is in negative association with the number of neutrophils in the airways of patients with asthma

**Fig. 1:**
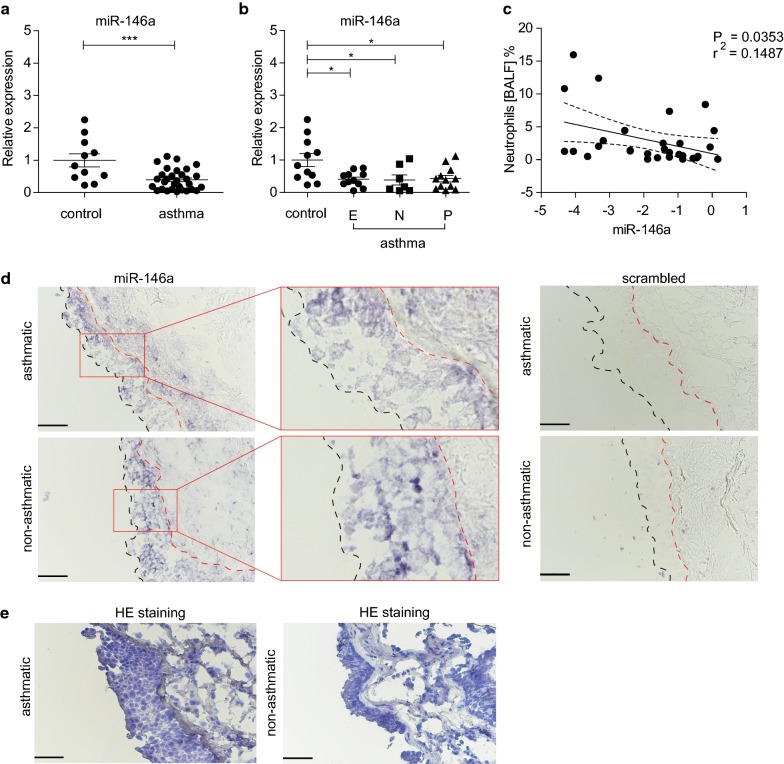
The expression of miR-146a is downregulated in epithelial cells from airway biopsies of patients with asthma. **a**–**c** Relative expression of miR-146a in bronchial brushing specimens from patients measured by RT-qPCR. Data represent mean ± SEM. **a** Unpaired t-test, ***P < 0.001. **b** Patients were categorized into inflammatory phenotypes based on BALF cell percentages: eosinophilic (E), > 2% of eosinophils and < 3% of neutrophils in BALF; neutrophilic (N), < 2% eosinophils and > 3% neutrophils in BALF; paucigranulocytic (P), < 2% of eosinophils and < 3% neutrophils in BALF). One-way ANOVA, *P < 0.05. **c** Linear regression analysis between miR-146a expression in asthmatic bronchial brushings (data presented on log2 scale) and neutrophil percentage among non-epithelial BALF cells. 95% confidence interval (CI) of the regression line is shown as dotted line. **d, e** 10 μm sections of frozen bronchial biopsy samples were subjected to in situ hybridization (**d**) or hematoxylin and eosin staining (**e**), bar = 50 μm. **d** Blue color shows the expression of miR-146a. Black dashed line indicates the outer border of the epithelium layer in the specimen. Red dashed line indicates the outer border of the basal lamina. One asthmatic and non-asthmatic sample representative of 3 and 2 subjects, respectively

To assess the role of miR-146a in association with asthma, we first examined miR-146a expression in bronchial brushing samples subjected to preserving conditions immediately after collection and later to simultaneous RNA purification. The bronchial brushing samples from asthma patients expressed significantly less miR-146a when compared with non-asthmatic controls (Fig. 1a). When patients were subgrouped based on inflammatory phenotypes, we observed miR-146a downregulation in airway epithelial cells from patients with eosinophilic, neutrophilic and paucigranulocytic asthma phenotype as compared to non-asthmatic controls (Fig. 1b). Linear regression analysis revealed a negative relationship between miR-146a expression in bronchial brushings and neutrophil cell counts in BALF in asthmatics (Fig. 1c). No association was found between the expression of miR-146a and eosinophil counts in the airways (Additional file 1: Figure S1A). To further analyze the expression and localization of miR-146a in human airways, we performed ISH on bronchial biopsy sections. The strongest miR-146a staining was confined to the bronchial (Fig. 1d) and bronchiolar epithelium (Additional file 1: Figure S1C), suggesting that in the lungs, miR-146a is mainly expressed in the bronchial epithelium. No significant difference in the expression level of miR-146a between the samples from asthma patients and controls were observed by ISH (Additional file 1: Figure S1B). Hematoxylin and eosin staining revealed an increased thickness of the basal lamina (Fig. 1e) and epithelial shedding (data not shown) in samples from the patients with asthma. In conclusion, we show that miR-146a is expressed in the epithelium layer of lung tissue and is reduced in airway epithelial cells from patients with asthma irrespective of lower airway inflammatory phenotype.

### The expression of miR-146a-influenced chemokines is increased in the airway epithelial cells from patients with neutrophilic asthma phenotype

**Fig. 2:**
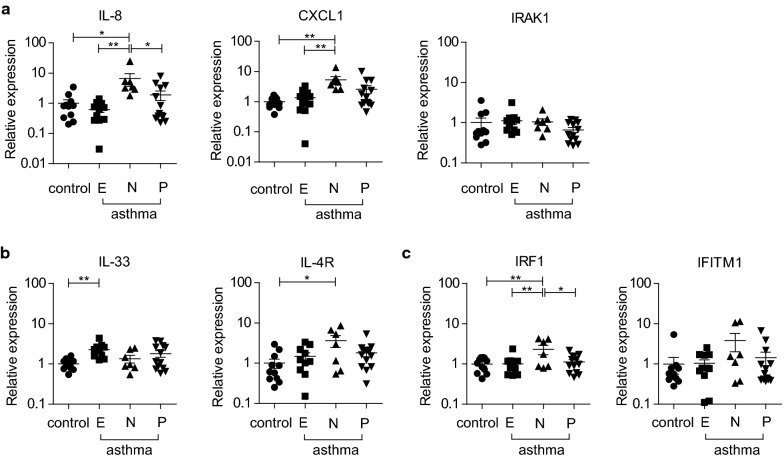
The expression of miR-146a-influenced chemokines is increased in brush biopsy airway epithelial cells from asthma patients with neutrophilic asthma phenotype. Relative mRNA expression of indicated miR-146a-influenced genes (**a**), genes associated with Th2 type responses (**b**) and interferon dependent genes (**c**) in bronchial brushing specimens from patients with asthma was measured by RT-qPCR and compared to the expression levels in the samples from control individuals. **a–c** Patients were categorized into inflammatory phenotypes based on BALF cell percentages: eosinophilic (E), > 2% of eosinophils and < 3% of neutrophils in BALF; neutrophilic (N), < 2% eosinophils and > 3% neutrophils in BALF; paucigranulocytic (P), < 2% of eosinophils and < 3% neutrophils in BALF). Data represent mean ± SEM. One-way ANOVA, *P < 0.05, **P < 0.01

As the miR-146a level was reduced in the airway epithelial cells from patients with asthma, we next analyzed mRNA expression of miR-146a indirect (CXCL1 and IL-8) [26, 28] and direct (IRAK1) [27] target genes in the same samples. In addition, to evaluate the relevance of our findings, we assessed the expression of genes previously associated with Th2 type immune responses, including IL-33 [32] and IL-4 receptor (IL-4R) [33], and the interferon-response genes associated with Th1 type immune responses, such as interferon regulatory factor 1 (IRF1) and IFITM1. Among these genes, only the expression of IL-33 was detected to be increased when all asthma patient samples were included to analysis (Additional file 1: Figure S1D). When the patients were subdivided by inflammatory phenotypes, the upregulated expression of miR-146a-influenced chemokines IL-8 and CXCL1 (Fig. 2a) as well as higher expression levels of IL-4R (Fig. 2b) and interferon response gene IRF1 (Fig. 2c) were detected in airway epithelial cells from patients with neutrophilic asthma. The expression of IL-33 was significantly higher in eosinophilic asthmatics when compared to non-asthmatics (Fig. 2b). miR-146a direct target IRAK1 did not differ between non-asthmatics and asthma patients in any analysis (Fig. 2a, Additional file 1: Figure S1D). Furthermore, linear regression analysis did not reveal any association between miR-146a expression and mRNA levels of tested indirect and direct genes (data not shown). Taken together, we detected that airway epithelial cells from asthma patients with neutrophilic phenotype had expected increased expression of IL-8 and CXCL1 while asthmatics with eosinophilic phenotype had an enhanced expression of IL-33.

### Pro-inflammatory cytokines induce the expression of miR-146a in HBECs

**Fig. 3:**
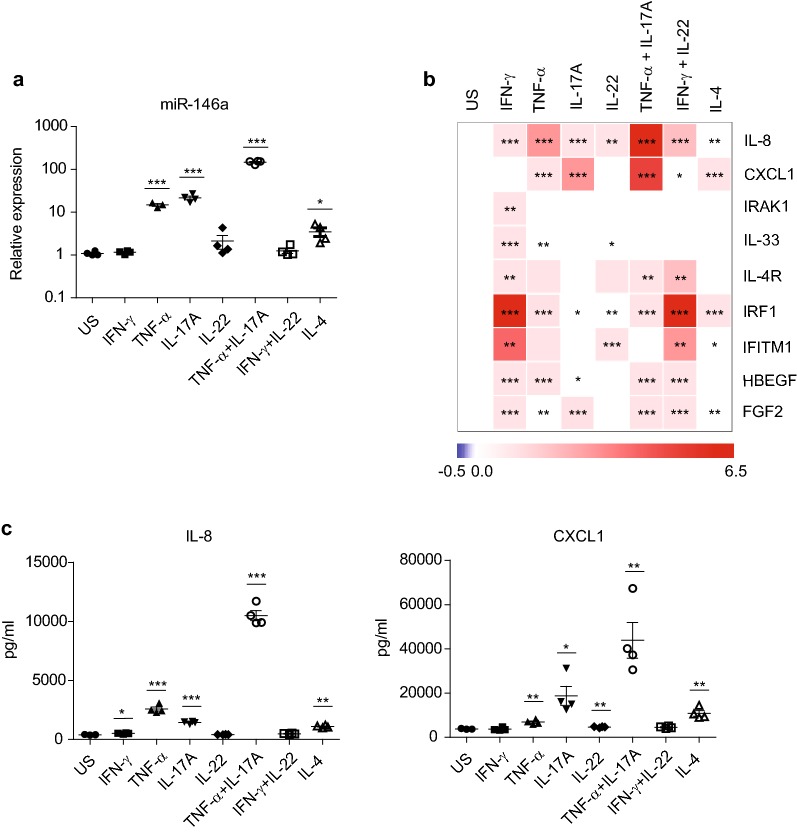
Pro-inflammatory cytokines induce the expression of miR-146a in HBECs. Cultured HBECs were stimulated with shown cytokines for 48 h or left unstimulated (US). **a** Relative expression of miR-146a expression was measured by RT-qPCR and is shown in comparison with US cells. **b** Heatmap of relative mRNA expression levels of indicated genes measured by RT-qPCR. Log2 values of fold changes were mean-centered for each gene separately. **c** IL-8 and CXCL1 protein levels from the supernatants of cytokine stimulated HBECs measured by ELISA. **a**, **c** Data represent mean ± SEM. **a–c** Unpaired t-test, *P < 0.05, **P < 0.01, ***P < 0.001

To understand how miR-146a expression is regulated in the airway epithelial cells, we stimulated primary cultured HBECs from healthy donors with cytokines associated with asthma pathogenesis, including IFN-γ, TNF-α, IL-17A, IL-22 and IL-4 [34, 35]. The expression of miR-146a was strongly induced by pro-inflammatory cytokines TNF-α and IL-17A, and with less extent by IL-4 (Fig. 3a). The highest increase in the expression of miR-146a was present in cells co-stimulated by TNF-α and IL-17A (Fig. 3a). No influence of IFN-γ and IL-22 on miR-146a level was detected. To evaluate the relevance of this result, we measured the expression of the same set of protein coding genes in stimulated HBECs that was analyzed in patients’ bronchial brushing samples. At the mRNA level, the expression of IL-8 was induced by all cytokines analyzed (Fig. 3b). However, at protein level, IL-8 was induced in response to TNF-α, IL-17A and IL-4 and most strongly by co-stimulation with TNF-α and IL-17A (Fig. 3c), which was similar to the changes in the expression of miR-146a (Fig. 3a) and CXCL1 (Fig. 3b, c). Out of the studied interferon response genes, IRF1 was highly induced in response to all cytokines analyzed, however, the expression of IFITM1 changed only in response to stimulation with IFN-γ, IL-22 or IL-4 (Fig. 3b). As chronic inflammation influnces cell proliferation associated with tissue remodeling, we also analyzed the expression of Heparin-binding EGF-like growth factor (HBEGF) and Fibroblast Growth Factor 2 (FGF2). As shown on Fig. 3b, HBEGF expression was induced in response to all studied pro-inflammatory cytokines except for IL-22 and IL-4 stimulation and FGF2 expression was increased by all cytokines but IL-22. The expression of Th2-type inflammatory genes IL-33 and IL-4R were also increased in response to IFN-γ stimulation (Fig. 3b), and to less extent by TNF-α. These data together demonstrate that several proinflammatory cytokines, including Th2-type cytokine IL-4, have capacity to stimulate the expression miR-146a in parallel with inflammation-associated mediators in primary HBECs.

### Overexpression of miR-146a leads to decreased expression of pro-inflammatory chemokines, diminished expression of airway remodeling associated growth factors and moderate increase in interferon response genes

**Fig. 4:**
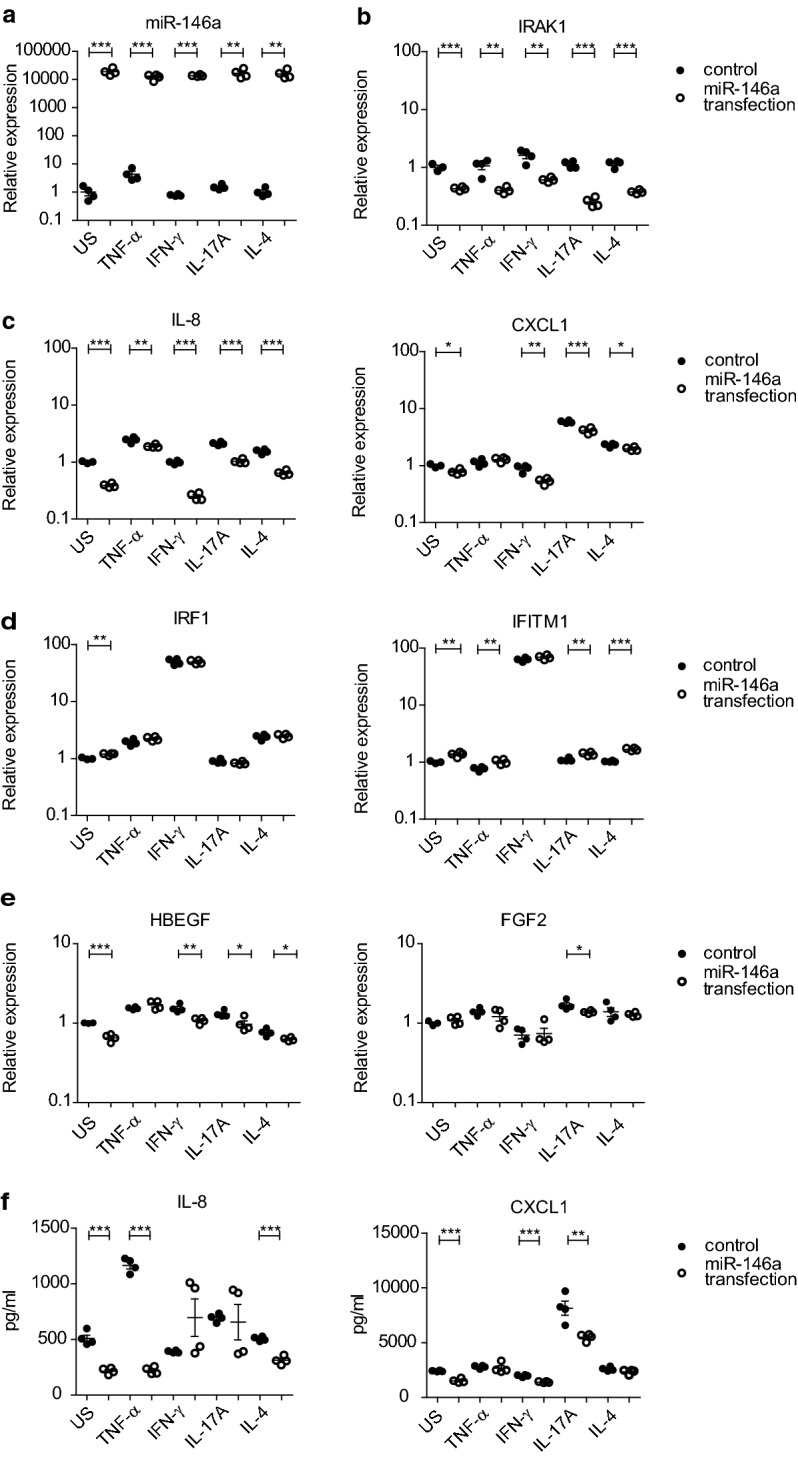
miR-146a inhibits the expression of pro-inflammatory chemokines IL-8 and CXCL1 and moderately upregulates interferon response genes. HBECs were transfected with miR-146a or control mimic and 24 h later stimulated with indicated cytokines or left unstimulated (US) for 48 h until harvesting. Relative miR-146a expression (**a**) and mRNA (**b–e**) expression was measured by RT-qPCR. (**f**) Protein levels of IL-8 and CXCL1 from the supernatants of transfected and stimulated HBECs were measured by ELISA. Data represent mean ± SEM. Unpaired t-test, *P < 0.05, **P < 0.01, ***P < 0.001

To better understand the function of miR-146a in the airway epithelial cells, we next transfected primary HBECs with miR-146a and control mimics and then stimulated the cells with pro-inflammatory cytokines (Fig. 4). Thereafter, miR-146a (Fig. 4a) and mRNA expression levels of the same genes that we analyzed in bronchial brushing samples and stimulated HBECs, were measured. As demonstrated in Fig. 4b, the expression of miR-146a direct target gene IRAK1 was strongly inhibited in miR-146a transfected cells in all used conditions. Concordantly, IL-8 mRNA was significantly decreased in miR-146a transfected cells in all conditions and CXCL1 was downregulated when stimulated with IFN-γ, TNF-α, IL-17A or IL-4 (Fig. 4c). In miR-146a transfected HBECs, the expression of IRF1 was induced in unstimulated cells and IFITM1 expression was increased in unstimulated conditions, as well as in cells stimulated with TNF-α, IL-17A or IL-4 (Fig. 4d). No effect on Th2 related genes IL-33 and IL-4R was observed by overexpression of miR-146a in HBECs (data not shown). The expression of growth factors related to tissue remodelling was also influenced by overexpression of miR-146a: HBEGF was reduced in most of the conditions and FGF2 in the presence of IL-17A (Fig. 4e). When transfected HBECs were costimulated with TNF-α and IL-17A, miR-146a overexpression led to the significant suppression of IL-8 and increase in IFITM1 expression (Additional file 1: Figure S2B, C). Decreased expression of IL-8 and CXCL1 in cells transfected with miR-146a was confirmed on protein level by measuring concentration of these chemokines in the cell culture supernatants (Fig. 4f and Additional file 1: Figure S2E). Together these data demonstrate that miR-146a has capacity to modulate inflammatory responses of HBECs via inhibiting the expression of pro-inflammatory chemokines IL-8 and CXCL1 and moderately upregulating the interferon response genes IFITM1 and IRF1.

### miR-146a inhibits the expression of secretable factors needed for neutrophil migration

**Fig. 5:**
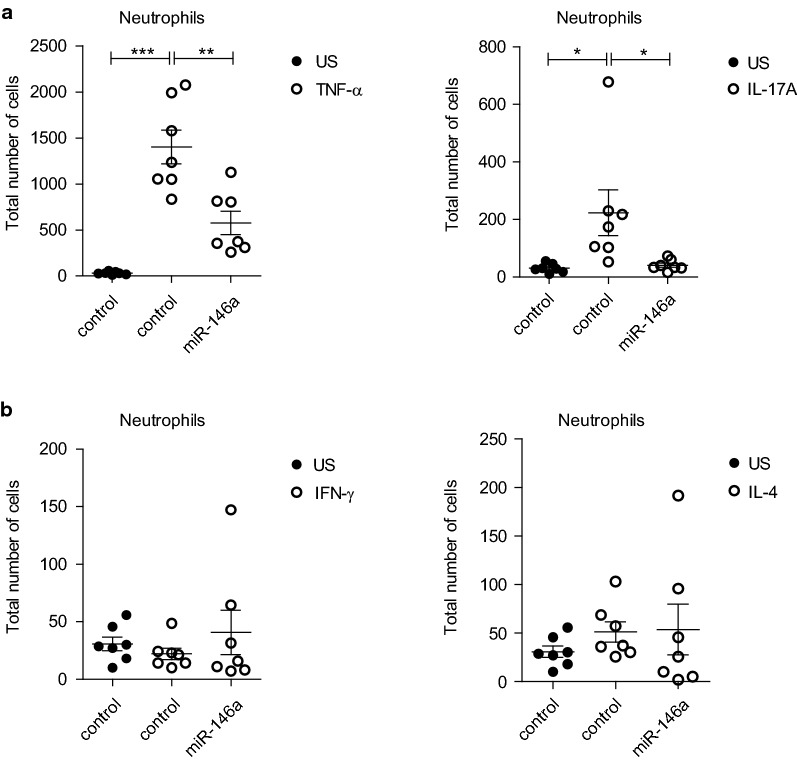
miR-146a inhibits the expression of secretable factors needed for neutrophil migration. Neutrophil chemotaxis assay was performed using supernatants of HBECs transfected with miR-146a or control mimic and stimulated with indicated cytokines as in Fig. 4. Migrated neutrophils were counted by flow cytometry. Data represent mean ± SEM. Unpaired t-test, *P < 0.05, **P < 0.01, ***P < 0.001

Since the expression of IL-8 and CXCL1, known to be chemotactic for neutrophils [36], were reduced in miR-146a transfected HBECs, we next studied whether miR-146a has an effect on neutrophil migration using cell supernatants from HBECs transfected with miR-146a or control mimic and stimulated with TNF-α, IFN-γ, IL-17A or IL-4. Stimulation of HBECs with TNF-α or IL-17A led to enhanced secretion of factors attracting neutrophils while overexpression of miR-146a in these conditions resulted in the suppression of neutrophil migration (Fig. 5a). The highest number of neutrophils was found in the supernatants from control mimic transfected and TNF-α + IL-17A stimulated HBECs, however, the effect of miR-146a was not significant in these conditions (Additional file 1: Figure S2F). The stimulation of HBECs with IFN-γ or IL-4 did not have an effect on the number of migrating neutrophils, neither was an influence of miR-146a in the presence of these cytokines observed (Fig. 5b). These results indicate that miR-146a has capacity to suppress the production of chemoattractants needed for neutrophil migration.

## Discussion

Asthma is a heterogeneous disorder, which is not always well controlled. Hence, there is an unmet need for better understanding of the mechanisms underlying the phenotypic diversity of asthma. In this study, we report significantly lower miR-146a expression in brushing biopsy airway epithelial cells from patients with asthma and a negative association between the reduced miR-146a expression and the number of neutrophils present in the airways. In the bronchial biopsy specimens, miR-146a was most highly expressed in the epithelium layer as determined by ISH. Further analyses showed expected excessive expression of neutrophil attracting chemokines IL-8 and CXCL1 in HBECs from the patients with neutrophilic asthma, while eosinophilic subgroup showed increased IL-33 expression. In cultured HBECs, the expression of miR-146a was induced in response to stimulation with pro-inflammatory cytokines characteristic of Th17 and Th2 driven inflammation. Overexpression of miR-146a in HBECs inhibited the expression of neutrophil attracting chemokines IL-8 and CXCL1 and reduced the number of migrating neutrophils in neutrophil chemotaxis assay. Together, our results indicate that reduced expression of miR-146a in airway epithelial cells from asthma patients may contribute to the development of neutrophilic phenotype of asthma.

Several studies have reported dysregulation of miRNAs in airway epithelial cells from asthmatics and suggest that these changes may contribute to the regulation of inflammatory responses and airway remodeling [37–40]. However, to our knowledge, our study is the first describing the downregulated expression of miR-146a in airway epithelial cells of asthma patients. This finding is consistent with a previous study showing that patients with severe asthma have lower expression of miR-146a in CD8^+^ and CD4^+^ T cells [41]. However, our observation is quite unusual as the expression of miR-146a has been more often reported to be increased in site of inflammation, for example in keratinocytes and in the skin of patients with atopic dermatitis and psoriasis [25, 26]. Using in situ hybridization, we observed that miR-146a is expressed in the lung epithelium and in bronchial, bronchiolar, and alveolar epithelium with no difference in the relative signal intensity of miR-146a between asthmatic patients and controls as analyzed by densitometry analysis. We propose that ISH using only a few samples is not sufficiently quantitative approach, however it is a suitable method to determine the expression location of miR-146a in lung epithelium. Interestingly, although miR-146a was downregulated in all asthma phenotypes, the linear regression analysis revealed a negative association between miR-146a levels in bronchial brushing samples and the number of neutrophils in BALF fluid, which prompted us to further address the question about the possible influence of miR-146a on the development of neutrophilic asthma phenotype.

To assess the relevance of the finding of the lower miR-146a expression in airway epithelial cells, we also tested the expression of selected protein coding genes. Increased expression of IL-8, CXCL1, IL-4R and IRF1 in patients with neutrophilic asthma and enhanced IL-33 in samples from eosinophilic patients confirmed phenotypic and endotypic similarity of these subgroups. As previous studies have shown miR-146a capacity to suppress IL-8 and CXCL1 in other cell types [28, 42], our results suggest that the lower expression of miR-146a may be linked to enhanced production of these chemokines by the airway epithelial cells in asthma. Although there was a negative association between the miR-146a levels and the number of neutrophils in BALF fluid, additional linear regression analysis did not reveal any association between relative miR-146a expression and mRNA levels of studied indirect and direct genes (data not shown). Also, we did not observe significant changes in IRAK1 levels in the samples from the patients with asthma regardless of the phenotype. We propose that miR-146a may act partially independently from IRAK1 in primary bronchial epithelial cells of patients with asthma, as previously demonstrated in primary human airway smooth muscle cells [43] and human alveolar A549 epithelial cell line [44]. Further studies would be needed to confirm this hypothesis and to delineate additional miR-146a direct targets in HBECs. Taken together, these data indicate that miR-146a may act through more complex network of genes not fully described by the current study.

To better understand the expression regulation of miR-146a, we also analyzed its level in HBECs stimulated with inflammatory cytokines. Similarly to keratinocytes [26], the expression of miR-146a was induced by TNF-α or IL-17A and additionally with IL-4 in HBECs. Even higher level of miR-146a was detected following treatment with combination of TNF-α and IL-17A, suggesting that these cytokines have a synergistic effect as in airway smooth muscle cells [45]. In line with previous publications [46–48], in our current study, HBECs from healthy donors expressed high amounts of IL-8 and CXCL1 in response to stimulation with TNF-α and IL-17A, as well as was the expression of interferon-regulated genes IFITM1 and IRF1 increased in response to IFN-γ. However, none of the used cytokines suppressed the expression of miR-146a leaving the question about downregulation mechanism in the patient samples unanswered. We propose that results from cell culture experiments using high concentrations of cytokines rather mimic processes of an acute inflammation, whereas patients with asthma had suffered from the disease on average for 15 years and therefore many changes in cells from bronchial brushing samples may represent characteristics of chronic inflammation. Furthermore, it is possible that the expression of miR-146a in HBECs of patients with asthma is decreased because of specific gene expression regulators, epigenetic and/or genetic differences. More specifically, microRNA expression relies on correct and functional microRNA biogenesis machinery, which can be disturbed in case of disease. For example, in lung cancer patients, a reduced expression of Dicer has been shown, which potentially might be responsible for decreased levels of different microRNAs in cancer cells [49]. Secondly, microRNA expression could be dysregulated due to epigenetic changes occuring during development of asthma as it is demonstrated for airway smooth muscle cells from asthmatic subjects [50]. Third possible explanation for downregulated miRNA expression could be genetic alterations. For example, in several types of lymphomas, the cause for dysregulated miR-15a and miR-16-1 is a deletion in 13q14 [51]. Interestingly, it has also been shown that in airways of healthy individuals, genes regulating cell cycle and immune system are less expressed with age and among them miR-146 family expression was significantly decreased, further indicating that miR-146a is one of the tightly regulated genes in the airways [52].

Several studies have demonstrated that miRNAs, such as miR-155 [7, 53, 54], miR-21 [55], miR-181 [56], and miR-223 [57], are involved in the regulation of airway inflammation. Although anti-inflammatory function of miR-146a has been demonstrated in various cell types, including bronchial epithelial cell lines [28, 30, 31], the function of miR-146a in primary HBECs had not been studied before. Our study shows that the expression of IL-8 and CXCL1 were decreased both on mRNA and protein level in HBECs transfected with miR-146a. We also demonstrate a strong reduction of IRAK1 and a moderate upregulation of IRF1 and IFITM1 mRNA expression in miR-146a-transfected HBECs. It should be noted that both the suppression of IL-8 and CXCL1 and increase of IRF1 and IFITM1 are most probably indirect effects of miR-146a. As no influence of miR-146a transfection on the expression of IL-33 and IL-4R was detected (data not shown), our results suggest that miR-146a does not influence Th2-type inflammatory responses in HBECs. However, our results revealed that miR-146a expression affects HBEGF expression in the presence of IFN-γ, TNF-α, IL-17A or IL-4 and FGF2 in the presence of IL-17A. Previous publications have shown upregulated expression of HBEGF in asthmatic tissues and its involvement in airway remodeling [58–60], indicating that reduced expression of miR-146a in airways of asthmatic patients may also be associated with increased airway remodeling, however, further investigation would be necessary to confirm this hypothesis.

Previous publications have demonstrated that HBECs express high amounts of IL-8 and CXCL1 in response to TNF-α and IL-17A in order to recruit neutrophils into the airways [46–48, 57]. Several other studies have shown that insufficient regulation of NF-κB signaling could be the origin of abnormally high expressions of IL-8 [61, 62] and CXCL1 [63], which in turn may be the potential cause of neutrophilic infiltration in the lower airways of asthma patients. As we observed a negative association between the relative miR-146a expression and the number of neutrophils present in the airways and reduced neutrophil migration towards the supernatants of HBECs transfected with miR-146a and stimulated with TNF-α or IL-17A, our data suggest that miR-146a is capable of inhibiting the production of neutrophil chemoattractants also in the airways of patients with asthma. These results together suggest that there is a strong link between reduced expression of miR-146a in airway epithelial cells in asthmatic patients and the development of neutrophilic phenotype of asthma.

In addition to data indicating that reduced expression of miR-146a may be linked to the recruitment of neutrophils in asthmatic airways, our study reveals other interesting asthma-related findings. In particular, we observed a tendency toward increased expression of IL-4R in airway epithelial cells from patients with neutrophilic asthma. As IL-4 binding with its receptor IL-4R mediates signaling during allergic inflammation and could rather be associated with airway eosinophilia, this result conveys certain controversies [64]. Nevertheless, Lavoie-Lamoureux et al. have demonstrated that IL-4 may also activate neutrophils by increasing the secretion of IL-8 and TNF-α and thereby providing a link between allergic, eosinophilic non-allergic and neutrophilic inflammation [65].

In summary, we show that miR-146a is expressed in the bronchial epithelium of the airways, whereas airway epithelial cells from patients with asthma express less miR-146a as compared to controls, whereas relative levels of miR-146a were detected to be in negative association with the number of neutrophils in BALF fluid. The cause of the reduced miR-146a expression in asthmatic airways is not known, however, it may lead to the increased neutrophil-attracting chemokines IL-8 and CXCL1 and thereby contribute to the development of neutrophilic phenotype of asthma. Our results provide a deeper understanding of inflammatory processes in asthma, and in long-term perspective, they might contribute to new potential options for therapeutic interventions.

## Conclusions

The current study indicates that reduced level of miR-146a in the airway epithelial cells of the patients with asthma may lead to the development of neutrophilic phenotype of asthma. miR-146a may have potential as a novel therapeutic molecule in the modulation of immune responses in asthma.

## Supplementary information

**Additional file 1.** Additional material and methods. **Figure S1.** The expression of miR-146a and selected genes in HBECs from bronchial brushings from patients with asthma. (A) Linear regression analysis between miR-146a expression in asthmatic bronchial brushings (data presented on log2 scale) and eosinophil % among non-epithelial BALF cells. 95% confidence interval (CI) is shown as dotted line. (B) An integrated density value of the signal per area of interest was obtained for 3 areas of bronchial mucosal biopsy samples from 2 non-asthmatic and 3 asthmatic patients. The relative intensity was calculated as percentage and was normalized to the average of control samples (100%). (C) In situ hybridization images of frozen lung tissue biopsy sections from asthmatic patient. Blue color shows the expression of miR-146a, bar = 50 μm. (D) Relative expression of indicated genes from bronchial brush biopsy samples of all included asthmatic patients was measured by RT-qPCR and compared to samples of non- asthmatic controls. Data represent mean ± SEM. Unpaired T-test, *P < 0.05. **Figure S2.** The expression of miR-146a and selected genes in HBECs transfected with miR-146a or control mimic (24 h) and stimulated for 48 h with TNF-α + IL-17A or left unstimulated (US). Relative miR-146a expression (A) and mRNA (B–D) expression was measured by RT-qPCR. (E) Protein levels of IL-8 and CXCL1 from the supernatants of transfected and stimulated HBECs were measured by ELISA. (F) Neutrophil chemotaxis assay was performed using supernatants of miRNA mimics transfected and TNF-α + IL-17A stimulated HBECs. Migrated neutrophils were counted by flow cytometry. Data represent mean ± SEM. Unpaired t-test, *P < 0.05, **P < 0.01, ***P < 0.001.

## Data Availability

Not applicable.
