# Supplementary material for: Reduced expression of miR-146a in human bronchial epithelial cells alters neutrophil migration
Source: Clin Transl Allergy. 2019 Nov 27;9:62. doi: 10.1186/s13601-019-0301-8 (PMC6880603; doi:10.1186/s13601-019-0301-8)
Supplement: Supplementary file 1 — Additional file 1. Additional material and methods. Figure S1. The expression of miR-146a and selected genes in HBECs from bronchial brushings from patients with asthma. (A) Linear regression analysis between miR-146a expression in asthmatic bronchial brushings (data presented on log2 scale) and eosinophil % among non-epithelial BALF cells. 95% confidence interval (CI) is shown as dotted line. (B) An integrated density value of the signal per area of interest was obtained for 3 areas of bronchial mucosal biopsy samples from 2 non-asthmatic and 3 asthmatic patients. The relative intensity was calculated as percentage and was normalized to the average of control samples (100%). (C) In situ hybridization images of frozen lung tissue biopsy sections from asthmatic patient. Blue color shows the expression of miR-146a, bar = 50 μm. (D) Relative expression of indicated genes from bronchial brush biopsy samples of all included asthmatic patients was measured by RT-qPCR and compared to samples of non- asthmatic controls. Data represent mean ± SEM. Unpaired T-test, *P < 0.05. Figure S2. The expression of miR-146a and selected genes in HBECs transfected with miR-146a or control mimic (24 h) and stimulated for 48 h with TNF-α + IL-17A or left unstimulated (US). Relative miR-146a expression (A) and mRNA (B–D) expression was measured by RT-qPCR. (E) Protein levels of IL-8 and CXCL1 from the supernatants of transfected and stimulated HBECs were measured by ELISA. (F) Neutrophil chemotaxis assay was performed using supernatants of miRNA mimics transfected and TNF-α + IL-17A stimulated HBECs. Migrated neutrophils were counted by flow cytometry. Data represent mean ± SEM. Unpaired t-test, *P < 0.05, **P < 0.01, ***P < 0.001. [file 13601_2019_301_MOESM1_ESM.pdf]

Additional file 1

Reduced expression of miR-146a in human bronchial epithelial cells alters neutrophil migration

Anet Kivihall[1], Alar Aab[1], Jerzy Soja[2], Krzysztof Sładek[2], Marek Sanak[2], Alan Altraja[3,4], Bogdan Jakiela[2], Grazyna Bochenek[2] & Ana Rebane[1]

[1] Institute of Biomedicine and Translational Medicine, University of Tartu, Estonia

[2] Department of Internal Medicine, Jagiellonian University Medical College, Krakow, Poland

[3] Department of Pulmonary Medicine, University of Tartu, Tartu, Estonia

[4] Lung Clinic of Tartu University Hospital, Tartu, Estonia

Correspondence: Anet Kivihall, Institute of Biomedicine and Translational Medicine, University of Tartu, Ravila 14B, 50414 Tartu, Estonia; E-mail: kivihall@ut.ee

Running title: miR-146a in human bronchial epithelial cells

Additional material and methods

1. Patient samples

The bronchial brushing samples were obtained from 30 adult patients with asthma from the Department of Pulmonology, the Jagiellonian University Medical College (Krakow, Poland). Asthma diagnosis was made according to Global Initiative for Asthma (GINA) guidelines by a pulmonologist or an allergologist. Specific information regarding patients with asthma is represented in Table 1. In addition, samples were collected from 11 age- and gender-matched non-asthmatic individuals (4 females) at age ranging between 29 and 75 years (mean age 52 years). All individuals studied were current nonsmokers. The bronchial brushing samples were used for total RNA isolation. Asthma patients were categorized into eosinophilic, neutrophilic and paucigranulocytic inflammatory phenotypes based on the number of granulocytes present in BALF (eosinophilic (E), >2% of eosinophils and <3% of neutrophils in BALF; neutrophilic (N), <2% eosinophils and >3% neutrophils in BALF; paucigranulocytic (P), <2% of eosinophils and <3% neutrophils in BALF). The cut-off values of granulocytes used for categorization were based on previous literature (1). Bronchial mucosal biopsy samples were collected from 3 adult patients with asthma and 2 non-asthmatic individuals from the Tartu University Hospital (Tartu, Estonia). The non-asthmatic patients underwent diagnostic bronchoscopy, but chronic airway disease was ruled out during further investigation. The collected lung biopsy samples were embedded into the Tissue-Tek (Thermo Scientific, Waltham, USA) and frozen. The frozen lung tissue biopsies were then cut into 10 μm sections and used for hematoxylin-eosin staining or *in situ* hybridization. The study was approved by the Ethics Committee of the Jagiellonian University Medical College and by the Research Ethics Committee of the University of Tartu. Each participant provided written informed consent.

2. Cell culture of normal human bronchial epithelial cells

Primary human bronchial epithelial cells were isolated from patients with asthma and control individuals' bronchial biopsies by initial short-term pronase (Roche, Basel, Switzerland) and DNase (Sigma-Aldrich) digestion. In total, cell lines were isolated from 1 asthma and 4 non-asthma subjects. Frozen stocks of passage 0 tracheobronchial epithelial cells were thawed and cultured in supplemented BEGM™ (Bronchial Epithelial Cell Growth Medium, Lonza, Basel, Switzerland) medium, containing bovine pituitary extract, insulin, hydrocortisone, gentamicin and amphotericin-B, retinoic acid, transferrin, triiodothyronine, epinephrine,

human epidermal growth factor (hEGF) according to manufacturers' recommendation and the cells were transferred to a cell culture incubator (at 37°C under 5% CO_2) for further experiments.

3. Stimulation of HBECs

For stimulation 3×10^4 cells per well were seeded on 12-well plate one day before the stimulation. For stimulations cytokines IFN-γ (final concentration 20 ng/ml, eBiosciences, USA), TNF-α (20 ng/ml, Biolegend, USA) and IL-17A (10 ng/ml, Peprotech, UK), IL-22 (20 ng/ml, Peprotech, UK), IL-4 (40 ng/ml, Peprotech, UK) were added to the cells. 48h after stimulations supernatant was collected from HBECs, 500 µl Qiazol (Qiagen, Germany) was added to stimulated cells and samples of cell lysate were kept at -20°C until total RNA isolation.

4. Transfection of HBECs

HBECs were seeded on 12-well plate at a density of 4×10^4 cells per well and 24h later miRIDIAN microRNA Mimic Negative Control #1 (Dharmacon™, USA) or miRIDIAN microRNA hsa-miR-146a-5p mimic (Dharmacon™, USA) were transfected using MIRFECT (RNAexact, Estonia) according to manufacturer's protocol. 24h after transfection HBECs were stimulated with cytokines IFN-γ, TNF-α, IL-17A and IL-4 as described in section 3.

5. RNA isolation

To isolate RNA from cytokine stimulated HBEC cells, Total RNA Mini kit (A&A Biotechnology, Poland) was used according to the manufacturer's instructions and to measure concentration of isolated RNA NanoDrop 2000c (Thermo Scientific, USA) spectrophotometer was used.

6. cDNA synthesis and RT-qPCR

To analyze miRNA expression from the HBECs of asthmatic and non-asthmatic patients TaqMan® MicroRNA Assays (Life Technologies, California, USA) and 5× HOT FIREPol® Probe qPCR Mix Plus (ROX) (Solis BioDyne, Tartu, Estonia) were used according to manufacturers' instructions. miRNA expression from cytokine stimulated HBECs was determined by using miScript II RT Kit, miScript SYBR Green PCR Kit and Hs_miR-146a_1 miScript Primer Assay (cat. MS00003535) by Qiagen according to the manufacturer's protocol. Each PCR reaction was performed in duplicates using a ViiA 7 Real-Time PCR system (Life Technologies, Carlsbad, California, USA). To normalize miRNA expression, let-7a and ΔΔCt calculation were used. To determine mRNA expression, cDNA was

synthesized using 200-500 ng of total RNA, oligo-dT (TAG Copenhagen, Denmark), RevertAid Reverse Transcriptase (Thermo Scientific) and RiboLock RNase Inhibitor (Thermo Scientific). For quantitative polymerase chain reaction 5x HOT FIREPol EvaGreen qPCR Supermix (Solis BioDyne, Estonia) and ViiA™ 7 machine (Applied Biosystems) were used. Target gene expression was normalized to EEF1A1 expression using ΔΔCt calculation. The following primers were used: CXCL1 fw 5' TTGCCTCAATCCTGCATCCC 3', CXCL1 rev 5' GGTCAGTTGGATTTGTCACTGT 3', EEF1A1 fw 5' CCACCTTTGGGTCGCTTTG CTGT 3', EEF1A1 rev 5' TGCCAGCTCCAGCAGCCTTCTT 3', IFITM1 fw 5' CAACACCCTCTTCTTGAACTGG 3', IFITM1 rev 5' GCCGAATACCAGTAACAGGATG 3', IL4R fw5' GCACCAAGTGGCACAACTCCT ACA 3', IL4R rev 5' AACAGGCAG ACGGCCAGGATGA 3', IL-8 fw 5' GCAGCTCTGTGTGAAGGTGCAGTT 3', IL-8 rev 5' TTCTGTGTTGGCGCAGTGT GGTC 3', IL-33 fw 5′ GACTCCTCCGAACACAGAGC 3′, IL-33 rev 5′ TGCTGTGGAAATTTTGTTGGTTG 3′, IRAK1 fw 5' CACCTTCAGCTTT GGGGTGGTAGTG 3', IRAK1 rev 5' CCAGCCTCCTCAGCCTCCTCT 3', IRF1 fw 5' CAACTTCCAGGTGTCACCCA 3', IRF1 rev 5' CGACTGCTCCAAGAGCTTCA 3', HBEGF fw 5' TTGTGCTCAAGGAATCGGCT 3', HBEGF rev 5' CAACTGGGGACGAAGGAGTC 3', FGF2 fw 5' AAAAACGGGGGCTTCTTCCT 3', FGF2 rev 5' TGTAGCTTGATGTGAGGGTCG 3'.

7. Protein quantification

To measure the levels of IL-8 and CXCL1 proteins in supernatants from HBECs, ELISA MAX™ Deluxe Set (BioLegend, 431504, the minimum detectable concentration of IL-8 for this set is 8 pg/mL) and human CXCL1/GRO alpha DuoSet ELISA (R&D Systems, DY275-05 with minimum standard concentration of 31,3 pg/mL) were used according to the manufacturers' instructions.

8. Neutrophil chemotaxis assay

Primary human neutrophils were isolated from a healthy donor's whole blood using gradient centrifugation on Ficoll-PaqueTM Plus (GE Healthcare, Chicago, IL, United States). To lyse erythrocytes, Red Blood Cell Lysis Buffer (Merck, Darmstadt, Germany) was used. Neutrophils were seeded at a density of 4×10^5 on ThinCert cell culture inserts (3-µm pore size) (Greiner Bio-One, Kremsmünster, Austria) and placed into 24 well plate. The outer chamber contained supernatants from HBECs transfected with miRNA mimics and stimulated with cytokines. 60 min after incubation at 37°C under 5% CO_2, the number of

neutrophils migrated from the insert into outer chamber supernatant was analyzed by using BD LSRFortessa (BD Biosciences, USA) cell analyzer.

9. *In situ* hybridization

In situ hybridization (ISH) was performed on 10 µm sections of frozen bronchial mucosal biopsy samples. For miR-146a detection miRCURY LNA miRNA ISH Buffer Set (FFPE) and hsa-miR-146a-5p miRCURY LNA miRNA Detection probe (final concentration 40nM) (cat. YD00619856) and control probemiRCURY scrambled ISH 49C (final concentration 40nM) (cat. YCD0074470-BCG) by Qiagen were used according to the manufacturer's protocol. Hybridization was performed at 49°C. Slides were incubated with alkaline phosphatase-conjugated sheep anti-DIG-AP (1:800, Roche, Switzerland) for 45 min at 30°C. To take pictures from the slides Leica DM5500 B microscope (Leica Microsystems) was used. Densitometry analysis of staining in *in situ* hybridization images were performed with ImageJ software (http://rsb.info.nih.gov/ij/index.html; public domain, NIH, USA) to obtain an integrated density value of the signal per area of interest and calculate the percentage value normalized to controls for each image.

10. Statistical analysis

For visualization and statistical analysis of RT-qPCR results, GraphPad Prism 5 (GraphPad Software Inc, USA) software and unpaired two-way Student's t-test, One-way ANOVA or linear regression analysis were used. The results were considered significant at * $P < 0.05$; ** $P < 0.01$; *** $P < 0.001$. To visualize RT-qPCR results as a heatmap, online matrix visualization and analysis software Morpheus by Broad Institute (https://software.broadinstitute.org/morpheus/) was used.

References

1. Heron M, Grutters JC, ten Dam-Molenkamp KM, Hijdra D, van Heugten-Roeling A, Claessen AME, Ruven HJT, van den Bosch JMM, van Velzen-Blad H. Bronchoalveolar lavage cell pattern from healthy human lung. *Clin Exp Immunol* 2012;167(3):523-531.

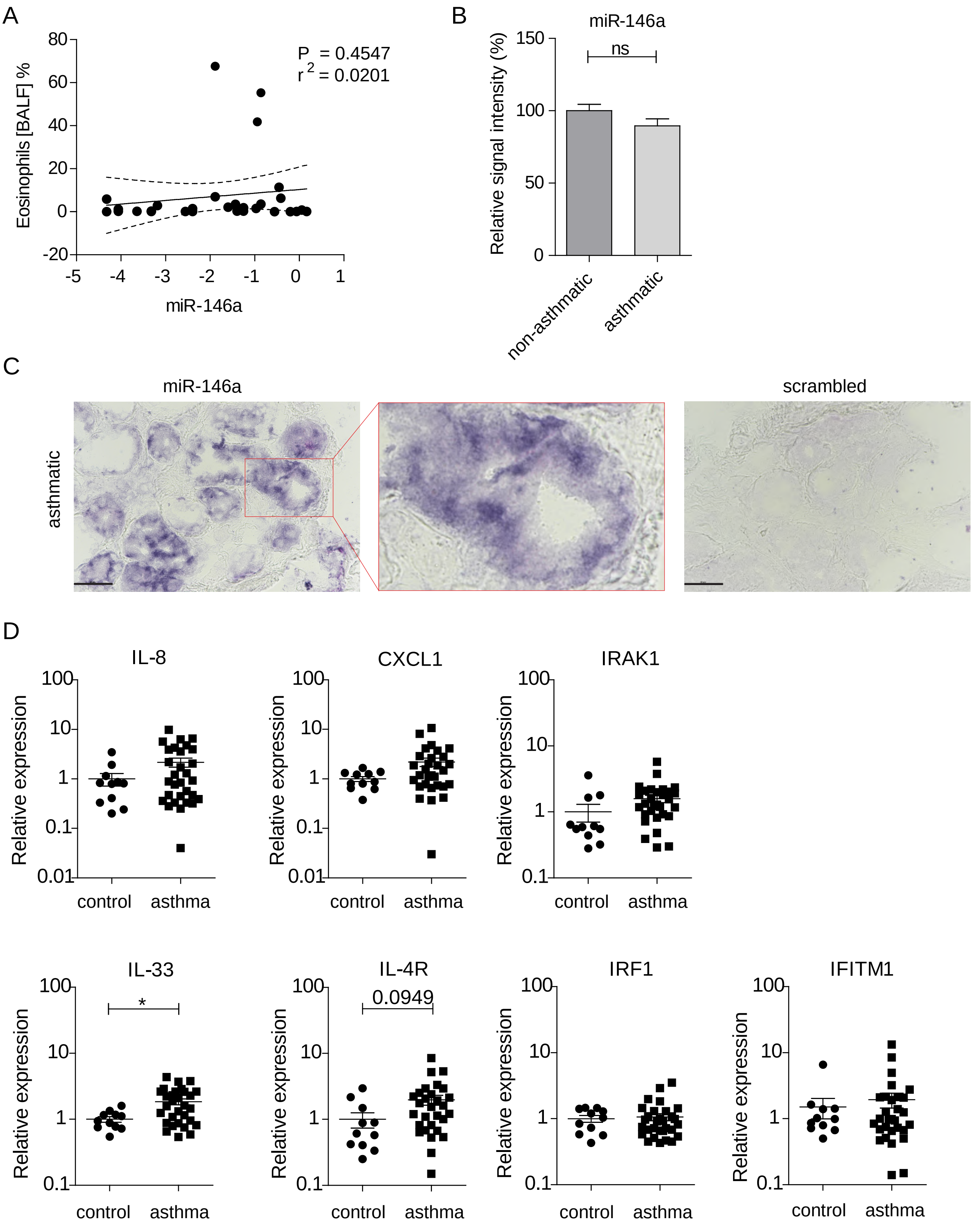

Figure S1. The expression of miR-146a and selected genes in HBECs from bronchial brushings from patients with asthma. (A) Linear regression analysis between miR-146a expression in asthmatic bronchial brushings (data presented on log2 scale) and eosinophil % among non-epithelial BALF cells. 95% confidence interval (CI) is shown as dotted line. (B) An integrated density value of the signal per area of interest was obtained for 3 areas of bronchial mucosal biopsy samples from 2 non-asthmatic and 3 asthmatic patients. The relative intensity was calculated as percentage and was normalized to the average of control samples (100%). (C) In situ hybridization images of frozen lung tissue biopsy sections from asthmatic patient. Blue color shows the expression of miR-146a, bar=50 μm. (D) Relative expression of indicated genes from bronchial brush biopsy samples of all included asthmatic patients was measured by RT-qPCR and compared to samples of non-asthmatic controls. Data represent mean ± SEM. Unpaired T-test, * $P < 0.05$.

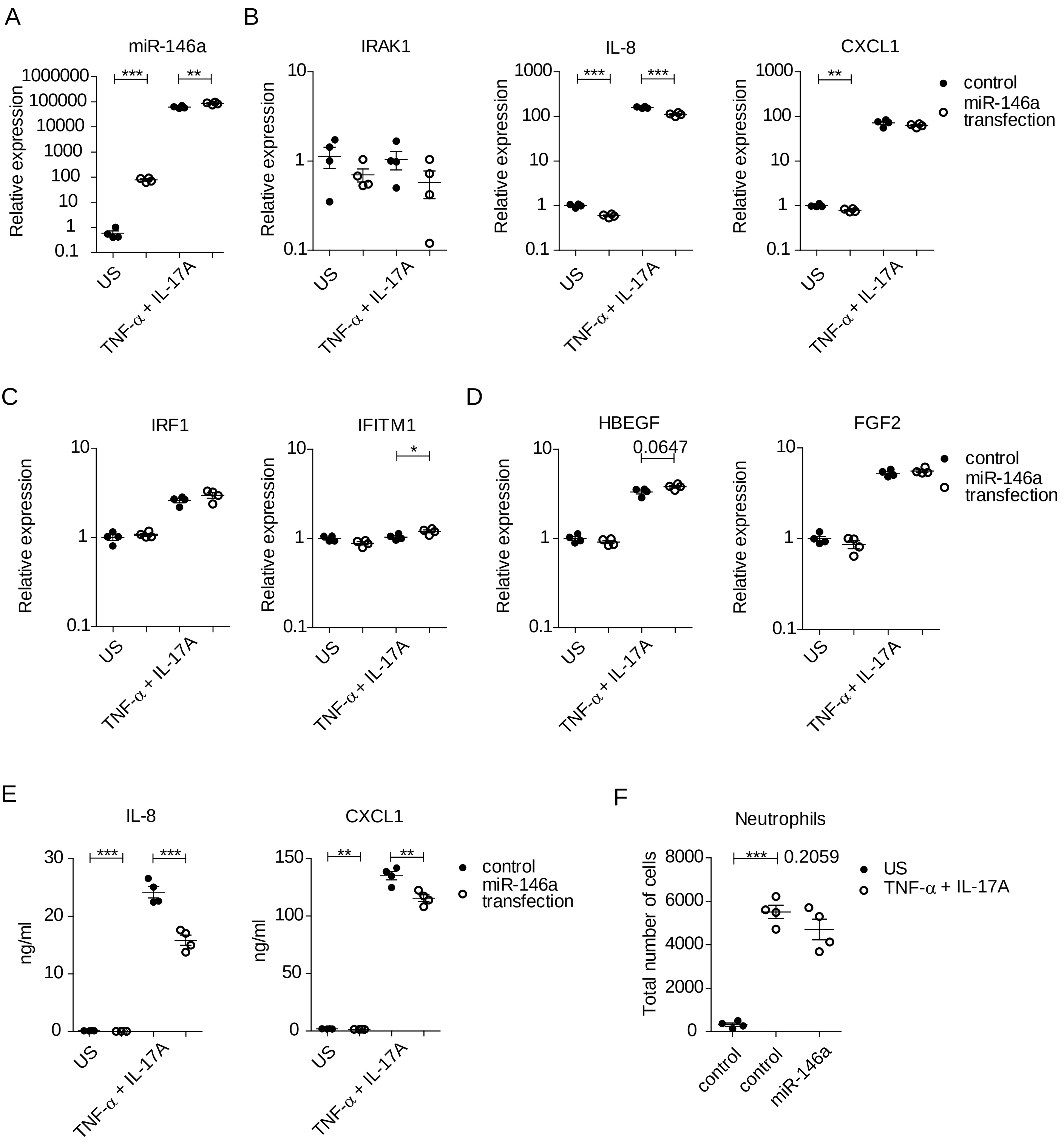

Figure S2. The expression of miR-146a and selected genes in HBECs transfected with miR-146a or control mimic (24h) and stimulated for 48h with TNF-α + IL-17A or left unstimulated (US). Relative miR-146a expression (A) and mRNA (B-D) expression was measured by RT-qPCR. (E) Protein levels of IL-8 and CXCL1 from the supernatants of transfected and stimulated HBECs were measured by ELISA. (F) Neutrophil chemotaxis assay was performed using supernatants of miRNA mimics transfected and TNF-α + IL-17A stimulated HBECs. Migrated neutrophils were counted by flow cytometry. Data represent mean ± SEM. Unpaired t-test, * $P < 0.05$, ** $P < 0.01$, *** $P < 0.001$.
